# Deep Learning‐Based Quantification of Spontaneous Pain Behaviours in Mice

**DOI:** 10.1002/ejp.70391

**Published:** 2026-09-23

**Authors:** Michele Zanoletti, Elisa Bellantoni, Lídia Pombo Gomes, Maria Luiza Quadros dos Santos, Daniel Souza Monteiro de Araújo, Gaetano De Siena, Sabrina Qader Kudsi, Romina Nassini, Marco Laurino, Francesco De Logu

**Affiliations:** ^1^ Institute of Clinical Physiology National Research Council Pisa Italy; ^2^ Department of Health Sciences Section of Clinical Pharmacology and Oncology University of Florence Florence Italy; ^3^ Graduate Program of Neurosciences, Institute of Biology Fluminense Federal University Rio de Janeiro Niteroi Brazil; ^4^ Graduate Program in Pharmacology Federal University of Santa Maria (UFSM) Santa Maria Rio Grande do Sul Brazil

## Abstract

**Background:**

Accurate evaluation of pain‐related behaviours in rodent models is crucial for elucidating pain mechanisms and assessing the efficacy of novel analgesic compounds. Traditional nociceptive assays primarily measure evoked responses, which have limited translational relevance since spontaneous pain is the predominant symptom in pain patients. Rodent models of spontaneous pain reveal innate behaviours, including paw licking and flinching, but their manual quantification is often subject to observer bias, is error‐prone and labor‐intensive.

**Methods:**

We have developed a deep‐learning‐based algorithm designed to automatically detect and quantify spontaneous pain behaviours in mice from standard 2D bottom‐up video recordings. Focusing on paw licking and flinching in the capsaicin pain model, and validated in the formalin test and a model of cancer‐evoked pain, our approach provides an objective, high‐throughput and reproducible alternative to traditional manual scoring.

**Results:**

The pipeline, combining DeepLabCut‐based keypoint tracking with a transformer classifier, achieved 92% balanced accuracy in identifying licking, flinching and baseline behaviours in the capsaicin model. Automated quantification showed an excellent correlation with expert human annotations (Pearson's *r* = 0.99), while frame‐by‐frame comparisons with blinded observers showed high agreement and minimal bias. Performance was maintained on an independently generated external dataset (Pearson's *r* = 0.98), supporting reproducibility across experimental settings. Validation in inflammatory and cancer pain models further demonstrated robust generalizability across distinct pathological conditions.

**Conclusion:**

By reducing observer‐dependent variability and capturing subtle temporal and spatial dynamics, this framework provides a standardized and accessible tool for the objective assessment of ongoing nociceptive responses, facilitating comparisons across distinct experimental models and institutional settings.

**Significance Statement:**

Our findings highlight the potential of deep learning to advance preclinical pain research by enabling standardized, unbiased and clinically relevant assessment of spontaneous pain in rodent models.

## 1 Introduction

Accurate assessment of acute and chronic pain in animal models remains a major challenge in preclinical research (Tappe‐Theodor and Kuner 2014). Pain is inherently subjective, and behavioural readouts are often influenced by operator‐dependent variables, leading to difficulties in standardization and reproducibility (Deuis et al. 2017; Urban et al. 2011). A wide range of behavioural assays has been developed to assess nociception in rodents (Le Bars et al. 2001; Turner et al. 2019). Traditional methods involve applying thermal, mechanical or chemical stimuli and measuring withdrawal responses as indicators of nociceptive thresholds (Barrot 2012; Lau et al. 2013). Different paradigms, including the von Frey test (Chaplan et al. 1994) and hot‐cold tests (Yalcin et al. 2009; Yoon et al. 1994) are widely used to evaluate hypersensitivity in pain models (Muley et al. 2016; Reitz et al. 2016). While highly informative, these assays primarily measure evoked responses, representing a translational limitation because only 15%–50% of patients experience evoked pain (Backonja and Stacey 2004), whereas nearly all report spontaneous pain (Freynhagen et al. 2011).

Spontaneous pain, which occurs in the absence of external stimuli, represents a more clinically relevant endpoint. In rodents, it is reflected by innate behaviours such as licking, biting, scratching, lifting or flinching (Mogil and Crager 2004). Among the available paradigms, the formalin test is considered a gold standard model of spontaneous inflammatory pain, producing a biphasic nociceptive response with an early phase driven by direct nociceptor activation, particularly through TRPA1 channels, followed by a later inflammatory phase associated with central sensitization (Dubuisson and Dennis 1977; McNamara et al. 2007; Tjolsen et al. 1992). Similar spontaneous behaviours are observed in capsaicin models via TRP vanilloid 1 (TRPV1) activation (Caterina et al. 1997). Facial expression analysis has emerged as a valuable approach for assessing spontaneous pain in rodents. The Mouse Grimace Scale (MGS), based on facial action units such as orbital tightening, nose bulge, ear position and whisker changes, provides a reliable measure of ongoing pain (Langford et al. 2010). However, manual MGS scoring is time‐consuming and subject to observer variability. To address these limitations, automated tools such as PainFace use deep learning to detect facial action units and generate standardized grimace scores from video recordings (McCoy et al. 2024).

Recent advances in computer vision and machine learning have transformed behavioural phenotyping in rodents by enabling automated and objective analysis of pain‐related behaviours. Deep‐learning‐based pose estimation frameworks, such as DeepLabCut (Mathis et al. 2018; von Ziegler et al. 2024) accurately track animal body parts and can be integrated with machine‐learning classifiers to detect behaviours including paw licking, biting, grooming and locomotor changes (Barkai et al. 2025; Wotton et al. 2020; Zhang et al. 2022). These approaches improve throughput and reproducibility while minimizing observer‐dependent variability. Existing machine‐learning methods have primarily focused on the detection of prolonged nocifensive behaviours, such as licking and biting (Wotton et al. 2020) which may exhibit limited sensitivity for capturing rapid and transient events such as paw flinching. Other comprehensive platforms utilize unsupervised clustering algorithms (e.g., Light Automated Pain Evaluator (Oswell et al. 2026)) to discover and analyse broad affective and motivational behavioural states over diverse timescales. Although highly effective for behavioural phenotyping, these unsupervised approaches are not inherently optimized for isolating short, high‐frequency nociceptive reflexes. Furthermore, recent approaches have incorporated complementary optical measurements to enhance behavioural resolution. These methods combine pose estimation with quantitative assessments of paw‐surface contact, luminance or depth‐related information, enabling the characterization of complex and three‐dimensional pain‐related behaviours (Barkai et al. 2025; Zhang et al. 2022). While providing highly detailed behavioural information, such systems typically rely on dedicated acquisition platforms and specialized hardware that may limit their accessibility and widespread implementation across laboratories.

To address these challenges, we developed a deep‐learning‐based pipeline for the automated detection and quantification of spontaneous pain‐related behaviours directly from standard bottom‐up video recordings. Our approach combines DeepLabCut‐based pose estimation with a custom transformer classifier that analyzes short temporal windows (0.2 s at 50 Hz), enabling detection of both sustained behaviours, such as licking, and highly dynamic responses, such as flinching. Unlike approaches that require dedicated optical hardware (Barkai et al. 2025; Zhang et al. 2022), our method operates using conventional video recordings, facilitating implementation across laboratories. The model was trained on the capsaicin‐induced pain paradigm and subsequently evaluated its performance in multiple independent pain models. In addition to acute inflammatory pain induced by formalin at different concentrations, we assessed the robustness of the algorithm in chronic pain conditions, including B16‐F10 melanoma‐induced cancer pain. These models are characterized by substantial alterations in posture and locomotion, providing a stringent test of classifier generalization.

By enabling standardized, high‐throughput quantification of spontaneous nocifensive behaviours across diverse pain states, this framework provides a practical and accessible tool for preclinical pain research and analgesic discovery.

## 2 Methods

### 2.1 Ethic

The research conducted complies with all relevant ethical regulations. Animal experiments were carried out in accordance with European Union (EU) guidelines for animal care procedures and the Italian legislation (DLgs 26/2014) application of the EU Directive 2010/63/EU. The study was approved by the National Committee for the Protection of Animals used for Scientific Purposes of the Italian Ministry of Health (permits no. 765/2019‐PR, 288/2021‐PR) and by the Ethics Committee on Animal Use of the Federal University Fluminense (CEUA/UFF; protocol no. 8817140126).

### 2.2 Animals

Male mice C57BL/6J (25–30 g, 6–8 weeks old) (Charles River, RRID:IMSR_JAX:000664 and Laboratory Animal Center (NAL) of the Federal University Fluminense (UFF), Niterói, Brazil) were used throughout. The group size of *n* = 6 animals for behavioural experiments was determined by sample size estimation using G*Power [v3.1; (Faul et al. 2007)] to detect size an effect in a post hoc test with type 1 and 2 error rates of 5% and 20%, respectively. Mice were allocated to vehicle or treatment groups using a randomization procedure (http://www.randomizer.org/). The assessors were blinded to the identity of the animals (allocation to treatment groups). None of the animals were excluded from the study. Behavioural studies followed Animal Research: Reporting of In Vivo Experiments (ARRIVE) (Kilkenny et al. 2010; McGrath and Lilley 2015). Animals were euthanized with inhaled CO_2_ plus 10%–50% O_2_. Mice were housed in a temperature‐ (20°C ± 2°C) and humidity‐controlled (50% ± 10%) vivarium (12 h dark/light cycle, free access to food and water, five animals per cage). At least 1 h before behavioural experiments, mice were acclimatized to the experimental room and their behaviour was evaluated between 9:00 am and 5:00 pm. All procedures were conducted following the current guidelines for laboratory animal care and the ethical guidelines for investigations of experimental pain in conscious animals set by the International Association for the Study of Pain (Zimmermann 1983).

### 2.3 Treatment Protocols

Mice received an intraplantar (i.pl., 0.5 nmol/20 μL/site) injection of capsaicin or its vehicle (0.25% ethanol in 0.9% NaCl), and one group received a specific antagonist of TRPV1, SB‐366791 (i.pl., 10 nmol/10 μL/site) or its vehicle (4% dimethyl sulfoxide (DMSO) in 0.9% NaCl) 30 min before the injection of capsaicin. In another set of experiments, mice received formalin (i.pl., 0.5, 2% and 4% 20 μL/site) or its vehicle (0.9% NaCl). Another group received a specific antagonist of TRPA1, A967079 (i.pl., 300 nmol/10 μL/site) or vehicle (2% DMSO and 2% Tween‐80 in 0.9% NaCl), 30 min before the injection of formalin.

### 2.4 Cancer Cell Inoculation

Naive (CRL‐6475; RRID:CVCL_0159 ATCC) B16‐F10 murine melanoma cells were cultured in DMEM containing 10% FBS and 1% penicillin–streptomycin (10,000 U/100 mg/mL) at 37°C with 5% CO_2_ in a humidified atmosphere and were used without further authentication. For inoculation, 20 μL of B16‐F10 melanoma (2 × 10^5^ cells) cells were suspended in PBS and injected into the plantar region of the mouse right hindpaw. Control groups (sham) were injected with 20 μL of PBS containing B16‐F10 melanoma (2 × 10^5^ cells) cells killed by quickly freezing and thawing them twice without cryoprotection.

### 2.5 Behavioural Assay

#### 2.5.1 Acute Nociception

Immediately after i.pl. injection, mice were placed inside a Plexiglas cylinder and a bottom‐up video was recorded for 10 min for capsaicin, 1 h for formalin and 10 min in mice at Day 14 after B16‐F10 melanoma cell inoculation. Nociceptive responses were quantified as the time the animals spent licking or flinching the injected paw and were annotated with millisecond precision. All videos were recorded in 1080 p resolution and analysed by investigators blinded to the experimental conditions using the developed machine‐learning model to classify and quantify spontaneous pain‐related behaviours automatically over the entire recording period.

### 2.6 Keypoints Detection

We employed DeepLabCut for body part tracking (version 2.3.11) (Mathis et al. 2018; Nath et al. 2019). A total of 900 frames were manually labelled from nine videos of nine different animals. To increase the dataset size, these frames were mirrored, yielding 1800 labelled frames. The videos were recorded in accordance with the described protocol, and four example frames are provided in the Video S1 and Table S1. Data were split into 95% for training and 5% for testing. We employed a ResNet‐50 backbone with pretrained weights. The batch size was set to 64 with an initial learning rate of 0.005, adjusted via a multistep schedule at 10 k (lr = 0.02), 430 k (lr = 0.002) and 730 k (lr = 0.001) iterations. The optimizer was stochastic gradient descent (SGD) with a weight decay of 0.0001. Data augmentation was performed using ‘imgaug’ with a global scale of 0.8, including contrast adjustments (CLAHE, histogram equalization) and augmentations such as embossing, rotation (25°) and cropping (0.4). The loss function incorporated location refinement with Huber loss (weight 0.05, standard deviation 7.2801). Training ran for 10^6^ iterations over 48 h on an NVIDIA GeForce RTX 3090 GPU and an Intel Core i7‐10700 CPU@2.90 GHz. Keypoints were defined across 18 anatomical locations, including nose, chin, throat, chest, stomach, two ventral points, proximal tail and both anterior and posterior paws (four points each).

### 2.7 Human Video Labelling and Behaviour Extraction

**FIGURE 1:**
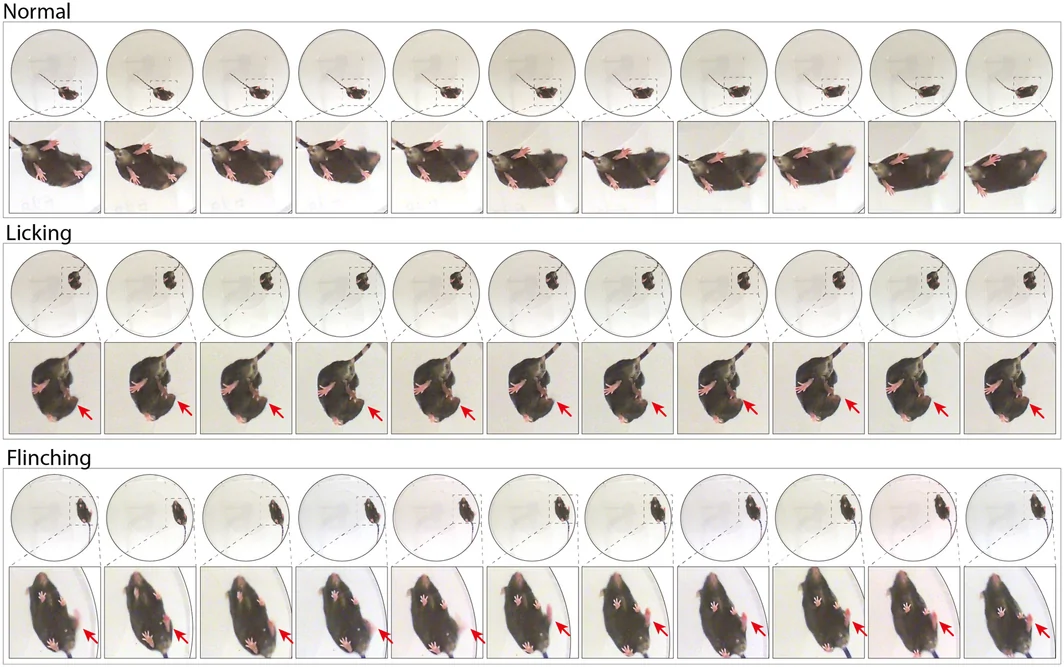
Representative examples of manually annotated pain‐related behaviours. Each row shows 11 consecutive frames illustrating normal behaviour, licking and flinching. Enlarged views highlight characteristic postures and movements, with arrows indicating paw‐directed licking or flinching responses.

For the subsequent behaviour classification stage, we compiled a new sequence‐based dataset. A total of 37 independent videos (recorded on mice injected with capsaicin) were analysed and mouse behaviours were manually annotated by expert human operators. Crucially, this dataset was strictly disjoint from the nine videos previously used for the keypoint detection stage (Section 2.6), ensuring no overlap of animals or frames. Furthermore, these 37 videos were completely independent from the experimental recordings used for the downstream biological testing of the pain models. The annotated behaviours included licking, flinching and baseline activity (Figure 1). We chose temporal windows of 0.2 s for the behavioural classification, corresponding to a minimum annotated duration of 11 frames, as videos were recorded at 50 Hz. Annotations were performed with millisecond precision, which was necessary due to the rapid dynamics of the flinching behaviour. In total, 44 baseline periods (mean duration: 37 s), 242 licking episodes (mean duration: 2.2 s) and 260 flinching episodes (mean duration: 0.7 s) were annotated. Because our model requires fixed‐length temporal sequences (11 frames) as input, extracting strictly non‐overlapping windows from a continuous behavioural episode would yield a very limited number of matrices for training. To overcome this and act as a form of temporal data augmentation, we applied a sliding window approach with a defined overlap (50% for licking and 80% for flinching) over the continuously labelled ground truth. By extracting multiple, shifted 11‐frame sequences from the same annotated event, this strategy provided the transformer with a significantly larger and richer dataset to learn the rapid temporal dynamics of these behaviours. After this procedure, approximately 1620 s of baseline behaviour, 1000 s of licking and 580 s of flinching were obtained. Licking and flinching segments were then manually reviewed, and ambiguous cases were discarded, resulting in 372 s of licking and 33 s of flinching. The substantial reduction in the flinching available time was due to the difficulty of obtaining representative movements within the 11‐frame window; unrepresentative sequences were excluded to avoid introducing noise into the dataset. To increase the dataset size and ensure spatial and structural invariance, specific data augmentation strategies were applied. For the baseline ‘normal’ class, frames were horizontally mirrored to introduce basic positional variance within the frame and double the dataset size. However, for ‘licking’ and ‘flinching’ behaviours (which are lateralized, paw‐related events characterized by a scarcity of examples) we applied a geometric augmentation strategy designed to simultaneously increase the dataset size by a factor of four and balance the paw anatomy. Specifically, we generated four geometric variations for each frame: the original image, a 180° rotation, a horizontal flip and a vertical flip. The original and 180° rotated frames preserve the animal's native anatomical left–right orientation, whereas the horizontal and vertical flips introduce a reflective transformation that inverts the left and right sides of the body. Consequently, 50% of the final augmented dataset contains the original paw anatomy and 50% contains the anatomically inverted anatomy, effectively eliminating any spatial or side‐specific bias at the dataset level. Nineteen videos were assigned to the training set and 18 to the test set, yielding the following total annotated durations: 3268 s (1835 training, 1433 test) for baseline behaviours, 1490 s (713 training, 777 test) for licking and 133 s (87 training, 46 test) for flinching.

### 2.8 Features Extraction and Behaviour Classification

For each frame, seven anatomical reference points were identified from the keypoint detection output: the midpoint between the nose and chin, the midpoint between the chest and abdomen, the proximal tail, left and right forepaws, and the left and right hindpaws (defined as the midpoint of the four respective hindpaw keypoints). The centre of mass was computed as the average position of these landmarks. To completely decouple the behaviour classification from the animal's absolute orientation within the camera's field of view, our downstream pipeline relies exclusively on animal‐centric features. Instead of analysing absolute screen coordinates, the transformer model evaluates four types of relative dynamic metrics: Euclidean distances between keypoints, visibility scores (derived from DeepLabCut likelihood values), instantaneous speeds (displacement between consecutive frames) and the cumulative number of directional changes. This strategic feature extraction forces the model to learn the actual physical dynamics of the nocifensive behaviour rather than memorizing spatial positioning. Feature extraction was tailored to each body part based on its relevance to the target behaviours. All four feature types were computed for the hindpaws, as they are directly involved in both licking and flinching movements; directional changes were particularly informative for detecting the characteristic rapid behaviour of hindpaw movements during flinching. For the forepaws, only velocities were extracted, as they provide information primarily through their movement (e.g., during walking) or relative stillness during other behaviours. For the chin, both velocity and visibility were included: visibility is frequently compromised during licking due to occlusion of the chin by the hindpaws, making it a discriminative feature for this behaviour. Additionally, for each frame, the distance between the centroid of all keypoints and its position in the first frame of the 11‐frame sequence was computed to capture the overall displacement of the animal within the field of view. Each classification unit consisted of an 11‐frame sequence (0.2 s at 50 fps) represented by an 11 × 13 feature matrix.

### 2.9 Behaviour Classification Model

We implemented a transformer‐based neural network to classify behaviours from 11‐frame sequences, each represented by a 13‐dimensional feature vector. For each sequence, these feature vectors were stacked to form the input matrix of dimensions 11 × 13, where rows correspond to consecutive frames and columns to the extracted features. Each frame‐level vector (dimension: 13) was linearly projected into a 32‐dimensional embedding space via a fully connected layer, with sinusoidal positional encoding for temporal ordering (Vaswani et al. 2023). The model architecture, alongside the training and evaluation pipelines, was implemented using PyTorch (version 1.12.1) in Python (version 3.8.16). The embedded sequences were processed by a four‐layer transformer encoder with multi‐head (four heads per layer) self‐attention and position‐wise feedforward networks (hidden dimension: 16). Residual connections and dropout (*p* = 0.3) were applied at each sub‐layer. Encoder outputs were aggregated via global average pooling and passed to a two‐layer feedforward classifier (32 units with ReLU and dropout, followed by a linear output layer for three behavioural classes). The model was trained (on the dataset obtained from videos of capsaicin‐injected mice, as previously described) for up to 100 epochs using cross‐entropy loss, the Adam optimizer (learning rate: 1e‐3 with plateau‐based scheduling) and weighted random sampling to address class imbalance. Performance was monitored on a validation set (20% of training data) with early stopping (patience: 10 epochs) based on balanced accuracy. The final model was retrained on the combined training, validation and test sets for the optimal number of epochs determined from the validation.

To interpret the decision‐making process of the transformer‐based classifier, we employed Shapley Additive exPlanations (SHAP, (Erion et al. 2021; Lundberg and Lee 2017)). SHAP values were calculated to estimate the impact of each extracted feature on the model's final class attribution across the different behavioural states. Furthermore, this post hoc explainability framework was explicitly applied to dissect the specific misclassification trajectory that exhibited the highest error rate within the model's confusion matrix.

### 2.10 New Video Labelling

**FIGURE 2:**
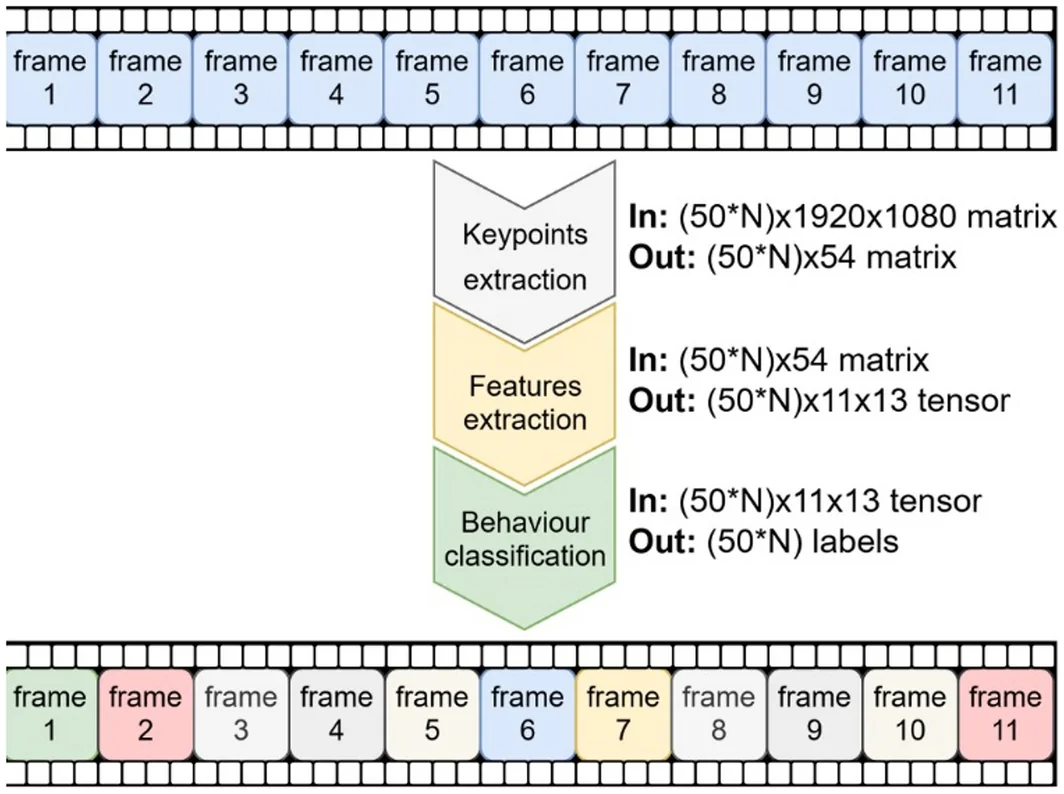
Overview of the video labeling pipeline. Each video, with duration *N* seconds, is sampled at 50 frames per second (50 × *N* total frames). Frames undergo keypoint extraction from the original 1920 × 1080 images, generating a (50 × *N*) × 54 matrix. Feature extraction transforms these data into a (50 × *N*) × 11 × 13 tensor, which is subsequently used for behaviour classification, yielding (50 × *N*) predicted labels.

The process for labelling a new video was structured as reported in Figure 2. Frames were first extracted at 50 frames per second. Each frame was then processed using the keypoints detection network. Features were subsequently computed for each frame, with the feature matrix for frame n constructed using a temporal window of 11 frames (from *n*–5 to *n*+5), corresponding to 0.2‐s segments. These feature matrices were then input to the transformer model to classify behaviours across the entire video. The final output consisted of a single‐frame labelling on the annotated video, as well as a file containing timestamps and the corresponding classifications. Following this labelling strategy, the trained pipeline was validated on six 1‐min videos to assess frame‐by‐frame accuracy and inter‐observer robustness. These videos, obtained from animals treated with capsaicin (three injected in the right paw and three in the left paw), were independently annotated at single‐frame resolution by three experienced operators blinded to the experimental conditions. Automated frame‐by‐frame annotations were then systematically compared against each human observer to evaluate the model's performance across individual scoring variations. Subsequently, the model was applied to full‐length recordings to automatically annotate behaviours in animals subjected to different pain models. These included 10‐min recordings for the capsaicin model (evaluating vehicle, capsaicin and capsaicin combined with a specific antagonist), 1‐h recordings for the formalin test across four conditions (baseline, vehicle, formalin and formalin combined with an antagonist) and 10‐min recordings in mice at Day 14 after B16‐F10 melanoma cell inoculation to evaluate cancer‐induced pain. For each condition, six videos were analysed.

### 2.11 External Inter‐Laboratory Validation

An external validation dataset was independently generated at a collaborating laboratory at the Institute of Biology, Fluminense Federal University, Niterói, Rio de Janeiro, Brazil. Mice (*n* = 6) received an intraplantar injection of capsaicin into the right hindpaw, were placed inside a Plexiglas cylinder and a bottom‐up video was recorded for 10 min. Behavioural responses were video‐recorded immediately after injection. Videos were subsequently analysed using the trained pipeline.

### 2.12 Materials

All reagents, capsaicin (#M2028), SB‐366791 (#S0441), formalin solution 10% (#HT501320) and A967079 (#SML0085) were from Merck Life Science S.r.l. (Milan, Italy).

### 2.13 Statistical Analysis

The results are expressed as the mean ± SEM. Before statistical significance analysis was performed, data were tested for normality using the Kolmogorov–Smirnov test and homogeneity using the Bartlett test. The reported group/sample size is the number of independent values. For multiple comparisons, a one‐way ANOVA followed by a post hoc Bonferroni's test was used. Statistical analyses were performed on raw data using GraphPad Prism 8 (GraphPad Software Inc.). *p*‐values less than 0.05 (*p* < 0.05) were considered significant. *p*‐values under 0.05, 0.01, 0.001 and 0.0001 are indicated. The statistical tests used and the group/sample size for each analysis are shown in the Figure legends.

## 3 Results

### 3.1 Keypoints Detection and Behaviour Classification

The models were trained on datasets obtained from mice subjected to capsaicin, vehicle or control conditions and tested on datasets from mice injected with capsaicin, a specific antagonist of capsaicin or vehicle, as well as from untreated controls (i.e., basal). Specifically, the keypoint detection model was trained using videos from four untreated control mice, two vehicle‐treated mice, one capsaicin‐treated mouse, one formalin‐treated mouse and one mouse treated with formalin plus a TRPA1 antagonist. Following this stage, we developed the sequence‐based behaviour‐classification model using a completely independent dataset. Importantly, this dataset was entirely distinct from that used for pose estimation, with no overlap in either videos or animals. The behaviour classification model was trained on 19 capsaicin videos and tested on an independent set of 18 additional capsaicin videos. Finally, its performance was further assessed by a frame‐by‐frame comparison using six additional independent capsaicin videos. The intraplantar injection of capsaicin induces a typical nociceptive response characterized by licking and shaking/flinching of the injected paw (Caterina et al. 2000). Licking refers to the animal repeatedly grooming or licking the affected paw to relieve discomfort or pain. Flinching involves rapid (typically high‐frequency) shaking movements characterized by repetitive paw twitching and elevation to reflect an acute withdrawal response to nociceptive stimulation (Barkai et al. 2025; Mogil et al. 2010). Together, these behaviours represent protective motor responses aimed at reducing further stimulation of peripheral nociceptors and serve as reliable indicators of pain intensity and duration in preclinical models. The trained models were subsequently validated on independent datasets, including recordings from formalin‐ and antagonist‐treated animals.

**FIGURE 3:**
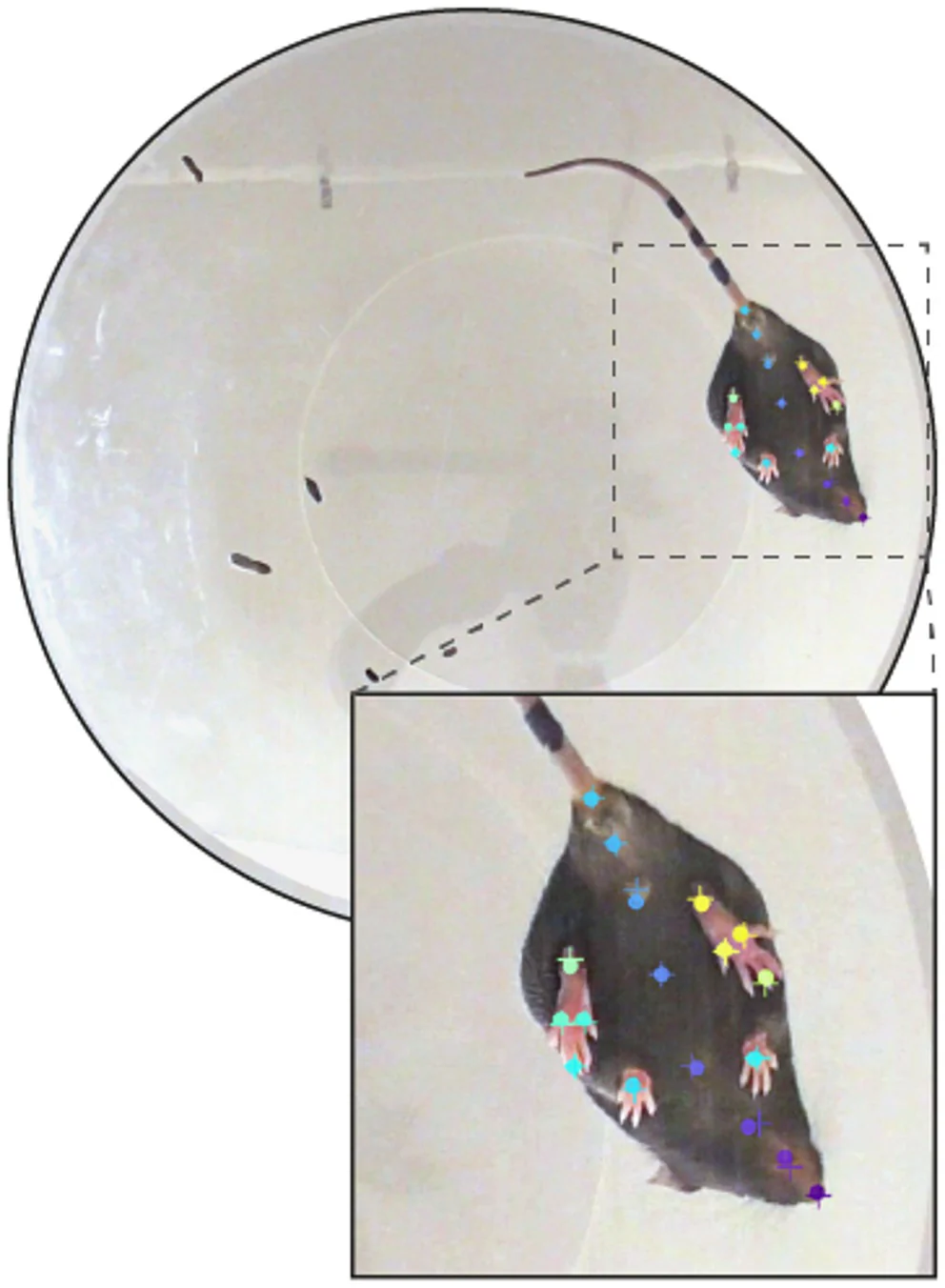
Representative labelled video frame from the training set illustrating manual annotations and model predictions. Crosses indicate human‐labelled keypoints, while dots represent model‐predicted keypoints.

**FIGURE 4:**
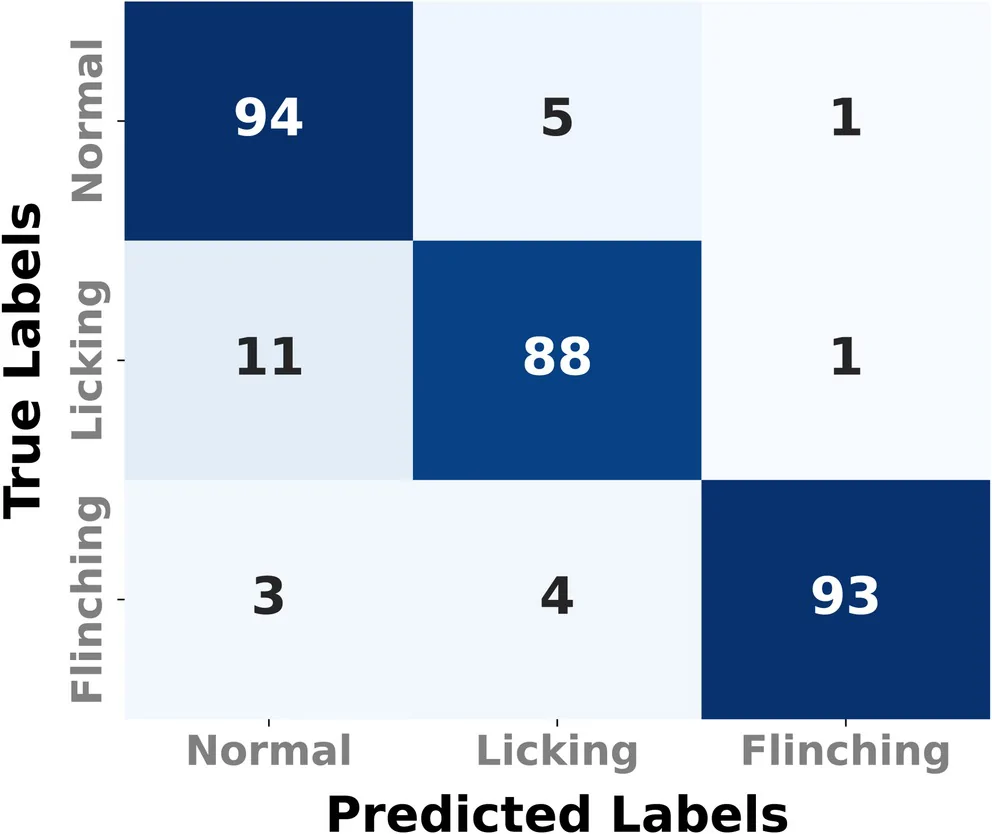
Confusion matrix showing the performance of the behaviour classification model on the test dataset for normal behaviour, licking, and flinching. Values represent the number of frames assigned to each class. The diagonal indicates correct classifications, while off‐diagonal entries represent misclassifications. The model achieved a balanced accuracy of 92%.

The trained network obtained an average error of 3.05 pixels on the training set and 7.02 pixels on the test set calculated across all keypoints. Given that the images had a resolution of 1920 × 1080 pixels, the average test error corresponds to less than 1% of the image size. This high spatial precision ensured accurate tracking of anatomical landmarks even during rapid nocifensive movements. An example of labelled frame from the training set is shown in Figure 3. Training was conducted for a maximum of 100 epochs, with early stopping applied according to validation performance, yielding optimal results at epoch 60. The final model achieved a training accuracy of 99.3% and a validation accuracy of 97.95%, with a balanced validation accuracy of 98.57%, indicating robust performance across all classes. On the test set, the model reached an overall accuracy of 92% and a balanced accuracy of 92%. Confusion matrix analysis showed consistent recognition across classes with minimal misclassification despite natural class imbalance (Figure 4). Notably, licking and flinching, that which represent rapid and transient events sometimes difficult to quantify manually, were accurately detected, confirming the model's sensitivity to the subtle temporal and spatial dynamics of spontaneous pain behaviours.

### 3.2 Frame‐By‐Frame Agreement

A total of 6 min from capsaicin‐injected mice, obtained from six different animals, was analysed at single‐frame resolution. To rigorously evaluate the agreement between model predictions and human expertise while accounting for inter‐observer variability, the automated model's outputs were systematically compared against frame‐by‐frame annotations from three independent blinded human scorers. A separate Bland–Altman analysis was performed for each observer to quantify bias and 95% limits of agreement (LoA) across behavioural classes (Table 1).

**TABLE 1:** Bland–Altman summary of model‐predicted versus human‐annotated behaviour durations for the three observers.

Observer	Behaviour	Bias (s)	LoA lower (s)	LoA upper (s)
1	Normal	−1.8	−5.5	2.0
1	Licking	−1.1	−3.7	1.5
1	Flinching	2.8	0.7	5.0
2	Normal	−9.5	−22.6	3.6
2	Licking	−2.1	−5.1	0.9
2	Flinching	11.6	−2.4	25.5
3	Normal	4.2	−2.2	10.6
3	Licking	−4.1	−8.9	0.7
3	Flinching	0.1	−3.5	3.7

*Note:* Bias indicates the average difference (human‐model) for each behaviour, while the 95% limits of agreement (LoA) indicate the interval within which most differences are expected to fall.

For observer 1, the model exhibited a tight agreement, showing a minor underestimation bias of −1.8 s for normal behaviour (LoA: −5.5 to 2.0 s) and −1.1 s for licking (LoA: −3.7 to 1.5 s), alongside a positive overestimation bias of 2.8 s for flinching (LoA: 0.7 to 5.0 s). Analysis against observer 2 revealed a wider scoring variance, reflecting a more conservative flinching annotation by this specific scorer; here, the model showed an underestimation bias of −9.5 s for normal behaviour (LoA: −22.6 to 3.6 s) and −2.1 s for licking (LoA: −5.1 to 0.9 s), with a positive bias of 11.6 s for flinching (LoA: −2.4 to 25.5 s). Conversely, comparison against observer 3 demonstrated a positive bias of 4.2 s for normal behaviour (LoA: −2.2 to 10.6 s), an underestimation bias of −4.1 s for licking (LoA: −8.9 to 0.7 s) and a near‐zero bias of −0.1 s for flinching (LoA: −3.7 to 3.5 s).

**FIGURE 5:**
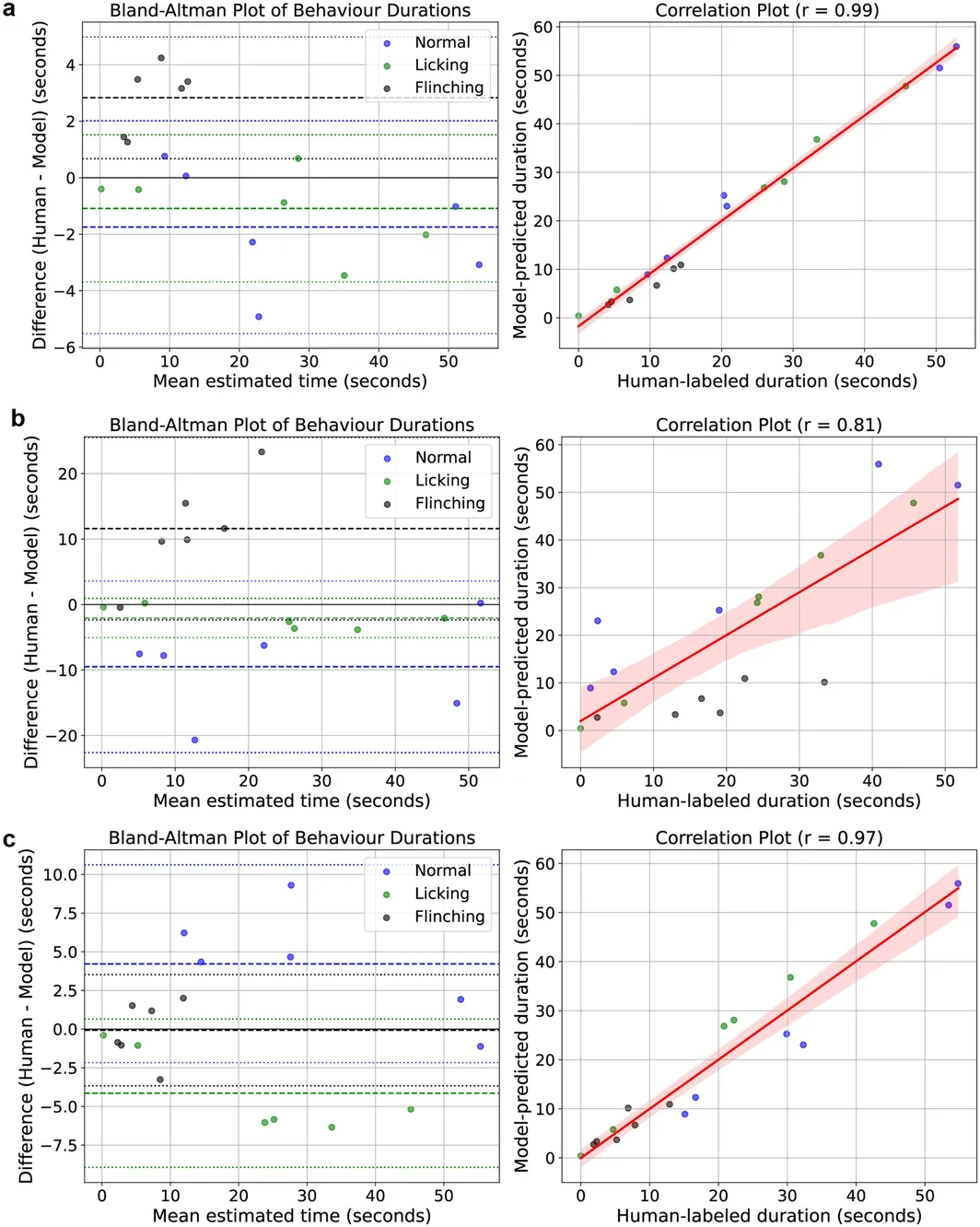
Agreement between human‐labelled and model‐predicted behavioural durations for Observer 1 (a), Observer 2 (b) and Observer 3 (c). For each observer, the left panel shows a Bland–Altman plot illustrating the difference between human‐labelled and model‐predicted durations as a function of their mean for normal behaviour, licking and flinching, whereas the right panel shows the correlation between human‐labelled and model‐predicted durations, including the linear regression and the 95% confidence interval. Strong Pearson correlations were observed for all three observers (Observer 1, a: *R* = 0.99; Observer 2, b: *R* = 0.81; Observer 3, c: *R* = 0.97).

**FIGURE 6:**
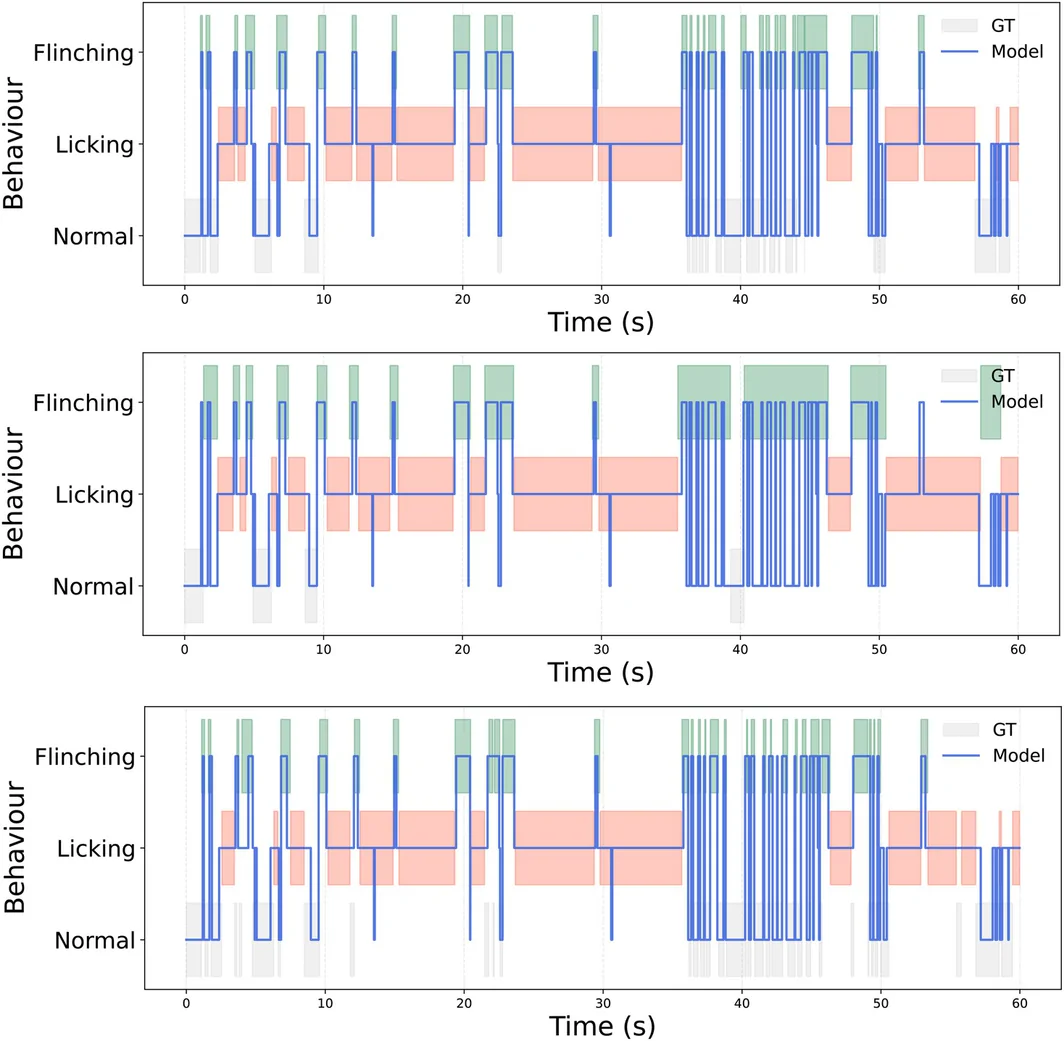
Frame‐by‐frame comparison between human observer annotations (ground truth, GT) and model predictions for pain‐related behaviours (Observer 1, a; Observer 2, b; Observer 3, c) over time. For each observer, shaded areas represent manually scored events for normal behaviour (grey), licking (pink) and flinching (green), whereas the blue line indicates the corresponding model predictions. Time is expressed in seconds.

Taken together, these multi‐observer metrics indicate that while human experts naturally exhibit subjective variations in parsing continuous behaviour templates, the automated pipeline consistently aligns within the distribution bounds of human scoring. Crucially, this robust frame‐by‐frame agreement was maintained across independent test videos encompassing both left‐ and right‐paw injection sites, supporting the overall spatial robustness of the pipeline against variations in animal orientation. Figure 5 reports the Bland–Altman agreement analysis for the three observers, while Figure 6 details their respective frame‐by‐frame temporal alignments. The labelled video is provided as Video S1.

**FIGURE 7:**
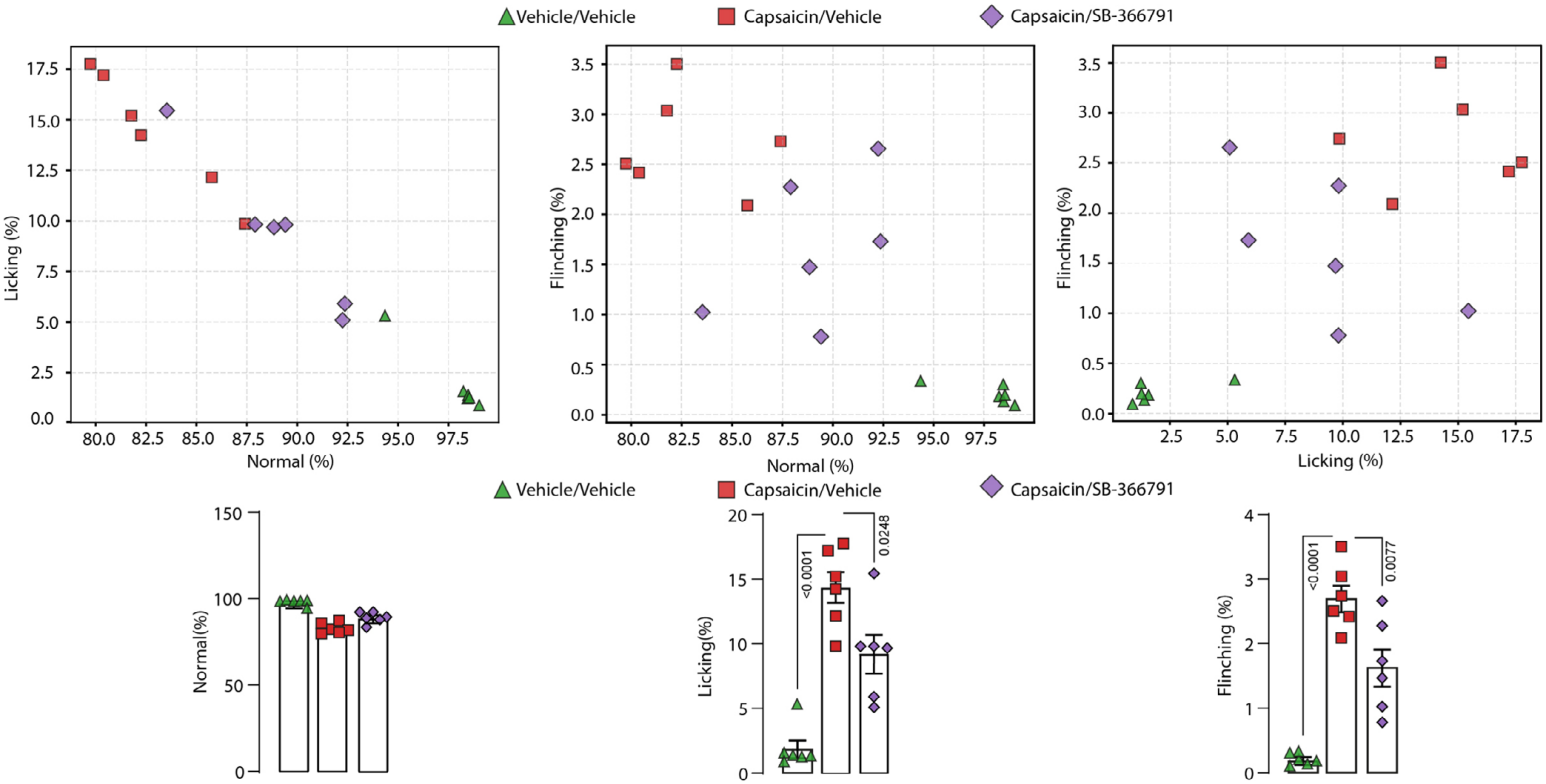
Scatter plots show the distribution of individual data points from 18 mice (*n* = 6 per group) in relation to cumulative pain‐related behaviours (normal behaviour, licking and flinching) detected by the model in the capsaicin test under vehicle treatment and in mice pretreated with SB‐366791. Bar graphs represent mean ± SEM. Statistical analysis was performed using one‐way ANOVA followed by Bonferroni's post hoc test.

The validation of the capsaicin‐induced nociceptive response was further confirmed by pharmacological intervention using a selective TRPV1 antagonist, SB‐366791. Local administration of the antagonist significantly reduced the characteristic pain‐related behaviours elicited by capsaicin. Automated analysis detected a marked decrease in the cumulative time spent in nociceptive behaviours compared to mice treated with capsaicin alone (Figure 7). Overall, the data further support the validity of the automated behavioural analysis system in reliably detecting reductions in the licking and flinching behaviours induced by TRPV1 antagonism in the capsaicin pain model.

### 3.3 Explainability and Feature Importance Analysis

**FIGURE 8:**
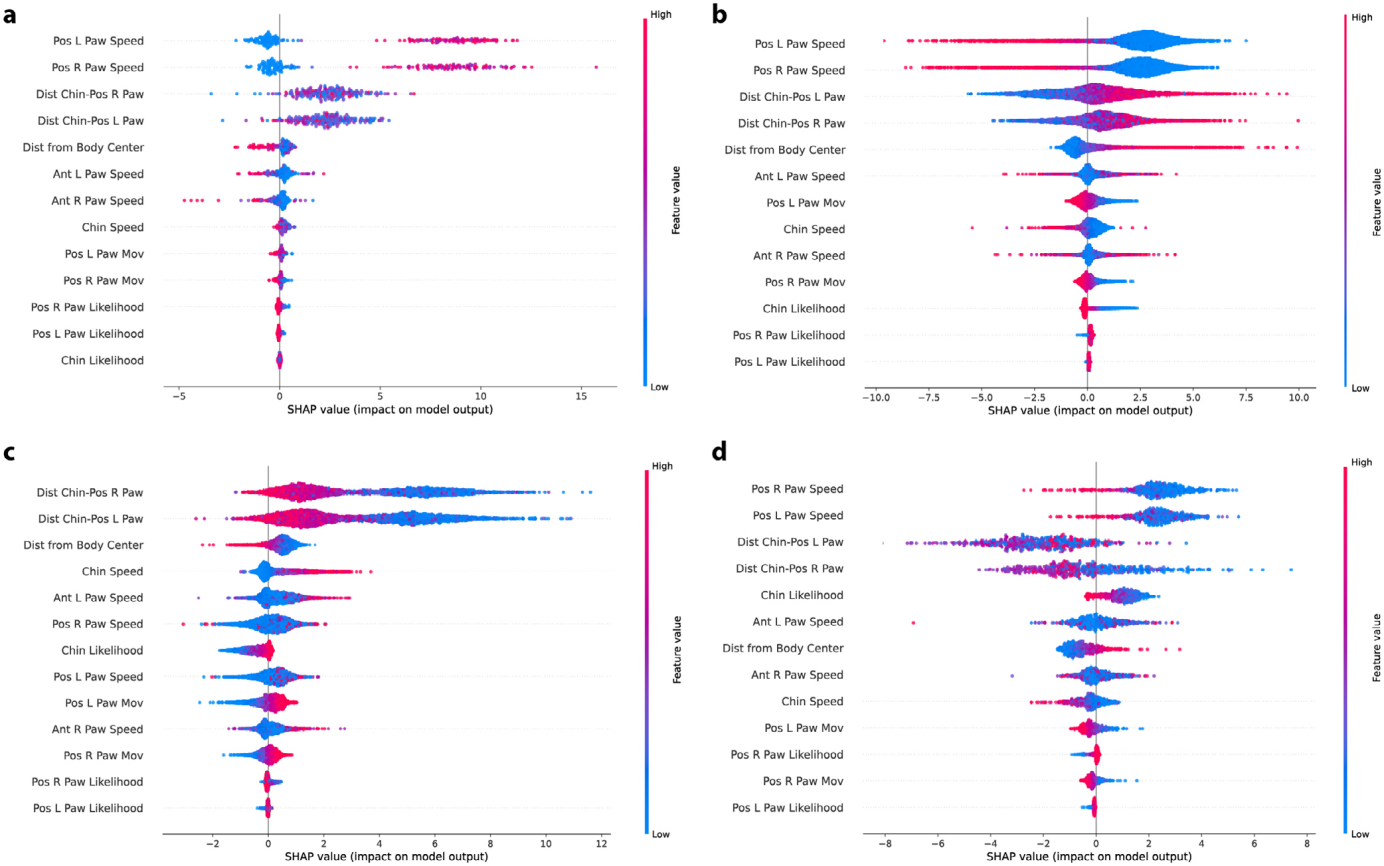
SHAP summary plots illustrating the contribution of individual kinematic and tracking features to the model predictions. (a) Correct flinching predictions, showing that classification is primarily driven by high instantaneous hindpaw speeds (*Pos L/R Paw Speed*, red dots on the right), confirming that predictions rely on true physical kinetics. (b) Correct baseline (normal) predictions, indicating that baseline behaviour is identified by low hindpaw speeds (blue dots for *Pos L/R Paw Speed* driving the prediction towards the right) and increased spatial separation between the snout and hindpaws (red dots for *Dist Chin‐Pos R/L Paw* on the right). (c) Correct licking predictions, demonstrating that classification is driven by compressed physical distances between the snout and the hindpaws (*Dist Chin‐Pos R/L Paw*, blue dots on the right). This confirms that the network successfully captures the characteristic condensed posture of the active licking response. (d) Primary misclassification trajectory (true licking predicted as normal), revealing that these errors are mainly associated with exceptionally low instantaneous hindpaw speeds (blue dots for *Pos R/L Paw Speed*), indicating that the model primarily struggles with static preparatory postures rather than the active licking movement. In all panels, the *x*‐axis represents the SHAP value, indicating the magnitude and direction of each feature's contribution to the prediction (positive values drive the model towards the predicted class), while dot colour represents the feature value, ranging from low (blue) to high (red).

To systematically evaluate the mechanistic basis of the transformer‐based classifier's predictions and uncover the kinematic features driving both accurate classification and systematic errors, we performed a post hoc explainability analysis using SHAP. For highly dynamic nocifensive responses, the model's correct identification of flinching was shown to be overwhelmingly governed by the high instantaneous speed of the hindpaws (Pos L/R Paw Speed; Figure 8a). Conversely, baseline behaviour (normal) was accurately identified when hindpaw speeds were low and the spatial separation between the chin and hindpaws (Dist Chin‐Pos L/R Paw) was maximized (Figure 8b). For accurate licking predictions, the model relies heavily on low physical distances between the chin and the hindpaws (Figure 8c). To resolve the limitations of the network, we utilized the SHAP framework to explain the specific misclassification category exhibiting the highest error rate in our confusion matrix: instances where true licking episodes were erroneously predicted as normal baseline behaviour. The analysis revealed that during these episodes, the classifier assigns to the ‘normal’ class primarily because the hindpaws exhibit exceptionally low instantaneous speeds (Pos R/L Paw Speed; Figure 8d). This indicates that the model primarily struggles with the static preparatory postures rather than failing to recognize the active licking kinematics.

### 3.4 External Inter‐Laboratory Validation

To assess the performance of the pipeline on data generated independently from those used for model development, we applied the trained model to an external dataset acquired at the Institute of Biology of the Fluminense Federal University (Niterói, Rio de Janeiro, Brazil). Videos were independently recorded using the local experimental and video‐acquisition setup and included mice receiving an intraplantar injection of capsaicin. None of these recordings was used during model development or training, and the pipeline was applied directly to the external dataset without site‐specific retraining, fine‐tuning or parameter optimization.

**FIGURE 9:**
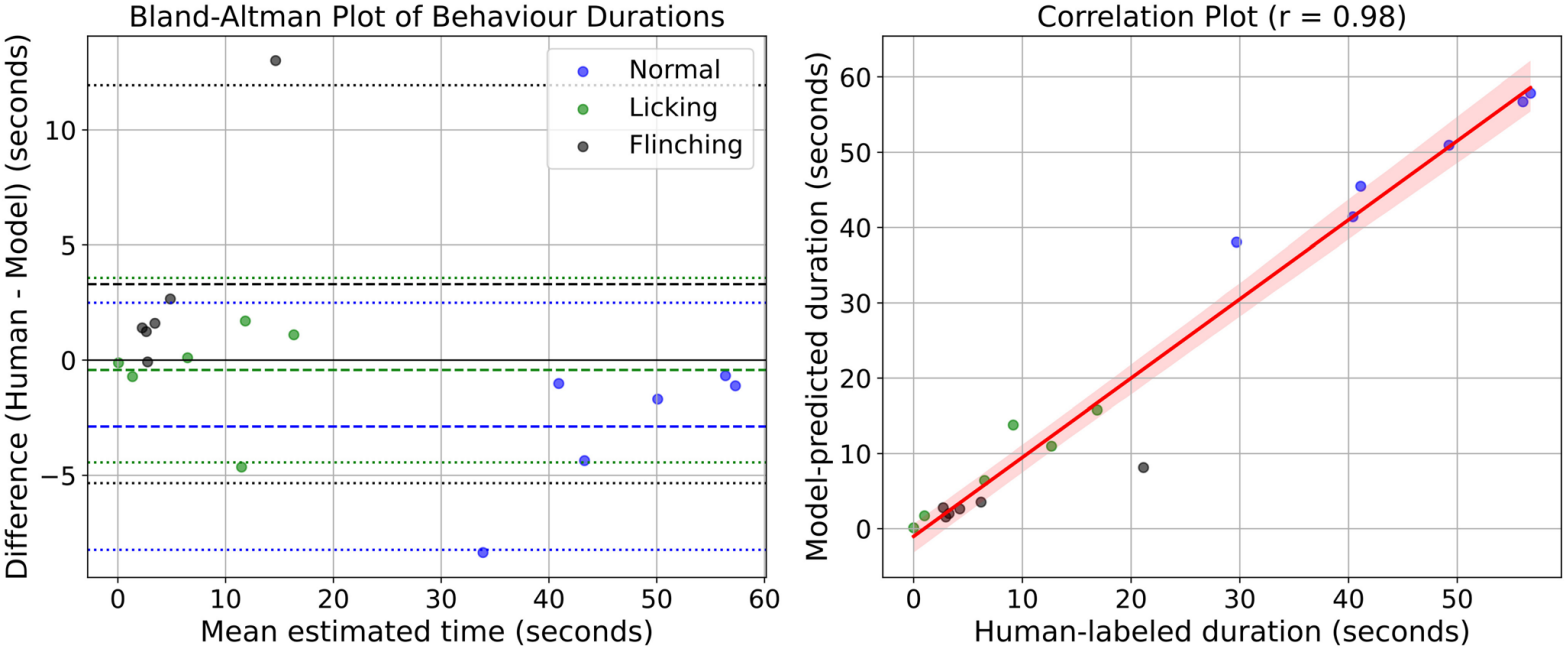
Agreement between human‐labelled and model‐predicted behavioural durations for the external inter‐laboratory validation dataset. The left panel shows a Bland–Altman plot illustrating the difference between human‐labelled and model‐predicted durations as a function of their mean for normal behaviour (blue), licking (green) and flinching (black) on videos generated at the external institution. The right panel shows the strong correlation (Pearson's *r* = 0.98) between human‐labelled and model‐predicted durations, including the linear regression (red line) and the 95% confidence interval (shaded red area).

When applied to the external dataset, the automated quantification demonstrated an excellent correlation with manual human scoring (Pearson's *r* = 0.98). Bland–Altman analysis further demonstrated close agreement between automated and manual measurements, with minimal systematic bias across behavioural categories: −2.9 s (95% LoA: −8.2 to 2.5 s) for normal baseline behaviour, −0.4 s (95% LoA: −4.4 to 3.6 s) for licking, and 3.3 s (95% LoA: −5.3 to 11.9 s) for flinching (Figure 9 and Table 2). Notably, despite differences in the experimental environment and video‐acquisition conditions, the pipeline preserved its ability to quantify the expected capsaicin‐evoked nocifensive response without any additional optimization. Together, these findings provide robust external inter‐laboratory validation of the trained pipeline and support its transferability and generalizability to independently generated behavioural video datasets.

**TABLE 2:** Bland–Altman summary of model‐predicted versus human‐annotated behaviour durations for the external validation dataset.

Behaviour	Bias (s)	LoA lower (s)	LoA upper (s)
Normal	−2.9	−8.2	2.5
Licking	−0.4	−4.4	3.6
Flinching	3.3	−5.3	11.9

*Note:* Bias indicates the average difference (human‐model) for each behaviour, while the 95% limits of agreement (LoA) indicate the interval within which most differences are expected to fall.

### 3.5 Formalin Pain Model

The pipeline was applied to video recordings from mice subjected to a typical inflammatory pain model induced by an intraplantar injection of formalin. Formalin administration produces a characteristic biphasic nocifensive response consisting primarily of licking, flinching and paw‐lifting behaviours. The first phase (Phase I; 0–10 min post‐injection) represents an intense acute reaction, followed by a brief quiescent period (interphase) and then a prolonged second phase (Phase II), which begins around 10–15 min post‐injection, peaks and gradually subsides. Notably, an elevated nocifensive response often persists for 60 min or longer after injection (Abbott et al. 1995; Sufka et al. 1998). This assay is widely used to assess non‐stimulus‐evoked spontaneous pain‐like behaviour and the sustained nature of the response provides valuable insights into the biological mechanisms underlying chronic pain. This response is mediated by the activation of TRPA1‐expressing sensory neurons and allows the quantification of spontaneous pain behaviours. Vehicle‐injected mice are used as controls to account for baseline motor activity and handling‐related responses.

**FIGURE 10:**
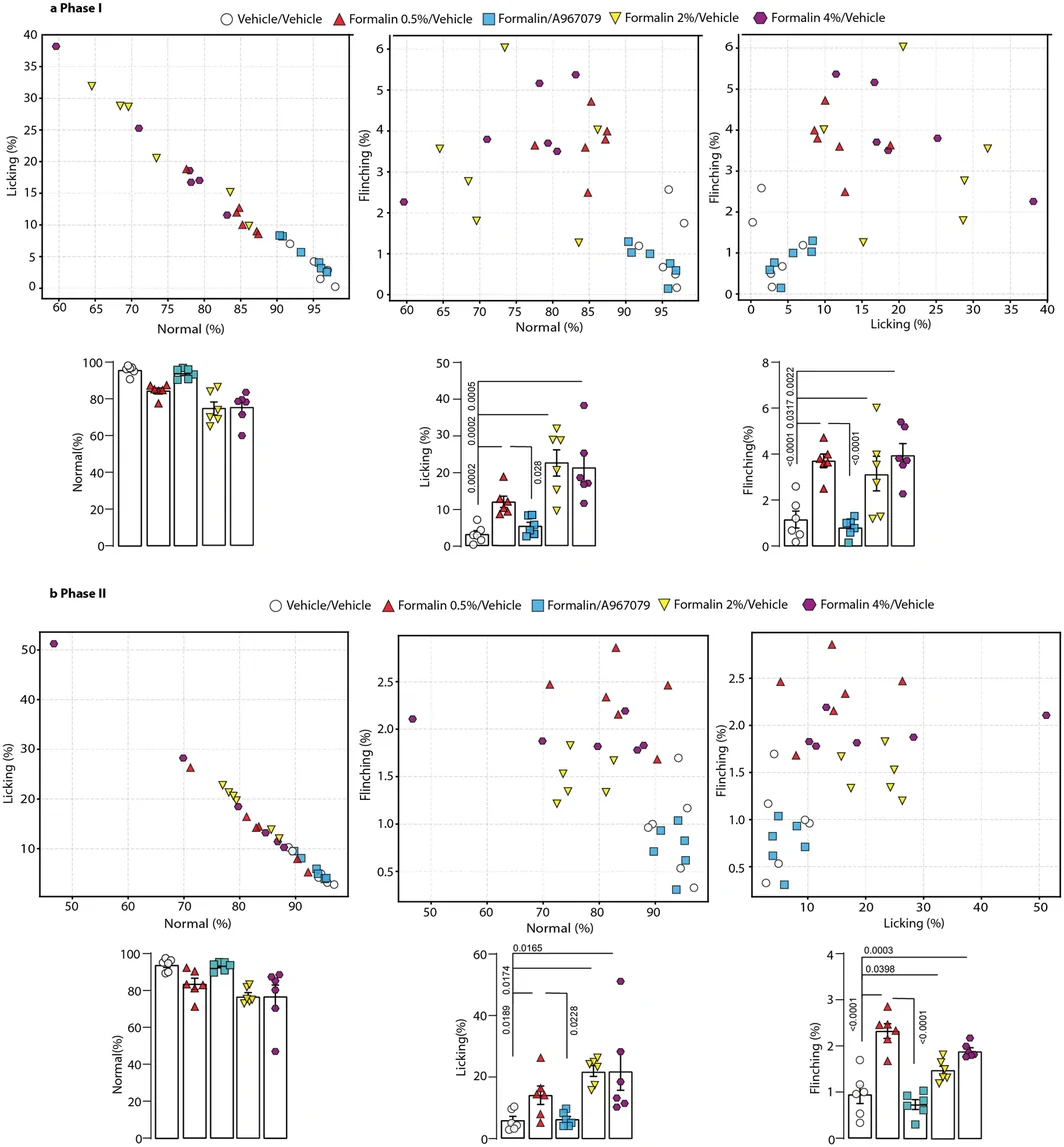
Scatter plots showing the distribution of individual mice (*n* = 6 per group; 30 mice total) according to the cumulative pain‐related behaviours automatically detected by the model during the formalin test. Relationships between normal behaviour, licking and flinching are shown for (a) Phase I (0–10 min) and (b) Phase II (11–60 min) in vehicle‐treated mice, formalin‐treated mice (0.5%, 2% and 4%), and mice pretreated with the TRPA1 antagonist A967079 before formalin administration. Bar graphs represent mean ± SEM. Statistical analysis was performed using one‐way ANOVA followed by Bonferroni's post hoc test.

During Phase I (0–10 min), intraplantar formalin elicited robust pain‐related behaviours, including licking, flinching and paw‐lifting compared with vehicle‐treated mice. Evaluation of three formalin concentrations (0.5%, 2% and 4%) and demonstrated that the automated pipeline was able to detect behavioural responses across different stimulus intensities (Figure 10a). A different group of mice receiving 0.5% formalin was treated with the TRPA1 antagonist A967079 to validate the ability of the pipeline to detect the pharmacological inhibition of pain behaviours. Pharmacological inhibition of TRPA1 markedly attenuated the characteristic flinching, licking and paw‐lifting behaviours induced by the intraplantar injection of 0.5% formalin (Figure 10a).

Pain‐related behaviours persisted throughout Phase II (10–60 min), reflecting the sustained inflammatory component of the response (Figure 10b). Behavioural responses remained readily detectable across the different formalin concentrations, whereas TRPA1 inhibition almost completely abolished these behaviours, confirming the critical contribution of TRPA1 signalling to the maintenance of prolonged inflammatory pain (Figure 10b). This approach enabled precise quantification of pain‐related behaviours and facilitated the identification of treatment‐dependent differences. The results obtained with the machine‐learning‐based analysis were comparable to those derived from traditional manual scoring methods, demonstrating its validity for behavioural assessment. Importantly, the use of machine learning increases the objectivity and reproducibility of data analysis, providing a valuable tool for large‐scale or longitudinal behavioural studies.

### 3.6 Cancer‐Induced Nociception

To further validate the robustness and translational applicability of the automated pipeline, we applied the model to a tumour‐induced pain paradigm based on intraplantar inoculation of B16‐F10 melanoma cells in C57BL/6J mice. This model has been previously used to investigate cancer‐evoked nociception, as progressive tumour growth (Day 10–14) within the confined plantar space promotes mechanical allodynia, inflammatory mediator release and neuro‐immune interactions that contribute to persistent spontaneous pain (De Logu et al. 2021).

**FIGURE 11:**
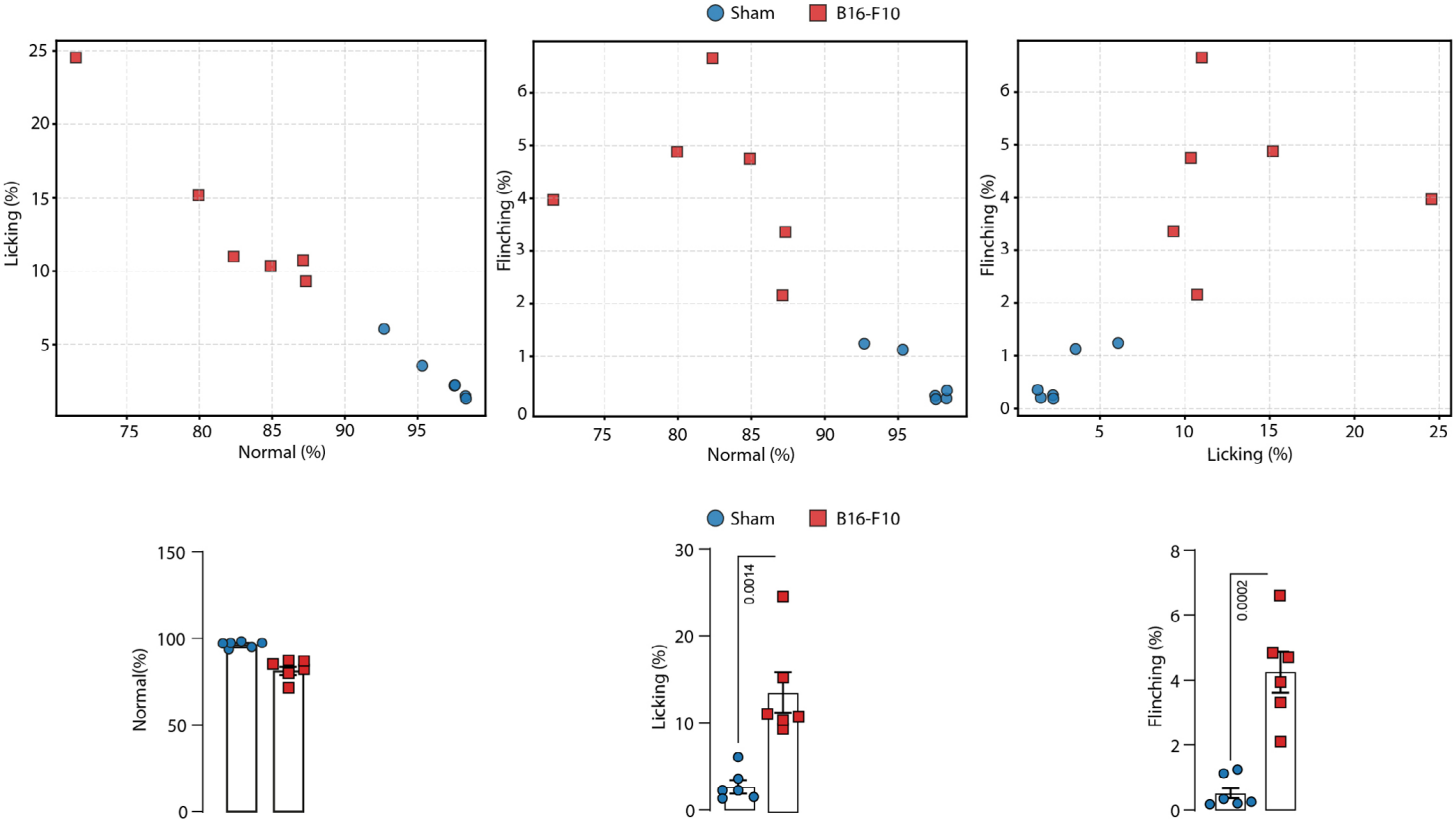
Scatter plots show the distribution of individual data points from 12 mice (*n* = 6 per group) in relation to cumulative pain‐related behaviours (normal behaviour, licking and flinching) detected by the model following hindpaw inoculation of B16‐F10 melanoma cells, compared with sham‐operated mice. Bar graphs represent mean ± SEM. Statistical analysis was performed using one‐way ANOVA followed by Bonferroni's post hoc test.

Using our automated behavioural analysis, we found that by Day 14 post‐inoculation, tumour‐bearing mice displayed a marked increase in spontaneous nocifensive behaviours, characterized by significantly enhanced licking and flinching of the ipsilateral paw compared to control animals (Figure 11). Importantly, the pipeline reliably detected these pain‐related behaviours in tumour‐bearing mice despite the locomotor alterations that may accompany tumour growth. Although gait was not quantitatively assessed in the present study, these results indicate that the classifier remains capable of identifying spontaneous nocifensive behaviours under chronic pathological pain conditions. Together, these findings indicate that the proposed automated approach enables accurate and objective quantification of cancer‐induced spontaneous nociception, providing a robust behavioural readout for studying tumour‐associated pain mechanisms and evaluating potential analgesic interventions.

## 4 Discussion

In this study, we developed and validated a deep‐learning‐based framework for the automated quantification of spontaneous pain behaviours in mice. Our pipeline integrates DeepLabCut‐based keypoint detection with a transformer‐based classification model that processes temporal sequences to assign behaviour labels at single‐frame resolution. The model was trained on capsaicin‐induced pain behaviours and achieving 92% balanced accuracy in distinguishing licking, flinching and baseline behaviours. Frame‐by‐frame validation against three independent expert observers demonstrated excellent agreement with minimal systematic bias despite the intrinsic variability of manual annotations. The algorithm was subsequently validated across independent pain models, demonstrating robust generalization. These results indicate that the proposed pipeline provides a robust and objective alternative to manual scoring, overcoming key limitations of current behavioural assays in preclinical pain research.

Traditional methods for pain assessment in rodents largely rely on evoked responses, such as withdrawal reflexes to thermal or mechanical stimuli (Barrot 2012; Chaplan et al. 1994; Yalcin et al. 2009; Yoon et al. 1994). While highly informative, these assays do not fully capture the spontaneous pain that represents the predominant symptom reported by patients in different painful conditions (Bennett 2012; Galer et al. 2000; Maier et al. 2010; Staud et al. 2007). Spontaneous nociceptive behaviours are more clinically relevant but have historically been difficult to assess objectively due to their rapid dynamics and the subjectivity of manual scoring. Our deep‐learning‐based approach directly addresses these limitations by enabling automated, reproducible and high‐throughput quantification of pain‐related behaviours. Specifically, flinching and licking behaviours, which are closely associated with ongoing pain states, were automatically detected and quantified, providing reliable indicators of spontaneous pain in rodents (Larson et al. 2019; Sufka et al. 1998). By detecting even subtle dynamics, the tool enhances the sensitivity of behavioural readouts and facilitates standardized comparisons across experimental conditions.

The present work introduces a targeted, complementary framework with distinct methodological advantages optimized specifically for quantifying rapid nocifensive movements. First, whereas recent state‐of‐the‐art platforms (Barkai et al. 2025; Zhang et al. 2022) achieve high precision by relying on highly specialized optical hardware to evaluate 3D paw elevation, our method achieves sensitive detection of these behaviours using purely standard, widely accessible 2D video recordings. Second, while comprehensive platforms utilizing unsupervised clustering (Oswell et al. 2026) excel at mapping broad behavioural structures and motivational states over extended timescales, our supervised, transformer‐based architecture was explicitly engineered to process short 0.2‐s temporal windows at 50 Hz. By intrinsically modelling the rapid temporal evolution of keypoint features within this brief window, our model is uniquely sensitive to isolating high‐frequency transient reflexes like flinching that might otherwise be merged or lost in broader clustering approaches.

Validation in the formalin model further underscores the utility of this approach. In the formalin test, the algorithm successfully detected nociceptive responses during both the early and late phases (McNamara et al. 2007) and revealed a significant attenuation of pain‐related behaviours following pharmacological inhibition of TRPA1. Importantly, these findings show that the classifier accurately captures both acute and sustained nociceptive behaviours, supporting its applicability across pain states with distinct temporal dynamics.

Importantly, the pipeline also proved effective in a model of cancer‐induced pain based on the intraplantar inoculation of B16‐F10 melanoma cells. Tumour‐bearing mice displayed enhanced spontaneous nocifensive behaviours at advanced stages of disease, which were reliably detected by the automated pipeline. Given that cancer‐related neuropathic pain is characterized not only by hypersensitivity to mechanical and thermal stimulation but also by prominent spontaneous pain, clinically presenting as burning, electric shock‐like, shooting, stabbing, lightning‐like or numbness‐like sensations (Wang et al. 2025), these findings highlight the translational relevance of the proposed approach and support its use for investigating tumour–nerve interactions and evaluating analgesic strategies in oncological settings. Although tumour growth may also affect posture and gait, quantitative gait analysis was beyond the scope of the present study. Future studies combining automated nociceptive behaviour detection with dedicated gait analysis could provide complementary insights into motor adaptations associated with chronic cancer pain.

Importantly, the generalizability of the pipeline was further supported by its application to an independent dataset generated at an external institution. Videos of capsaicin‐treated mice were independently acquired by a collaborating laboratory using its own experimental and recording setup and were subsequently analysed with our trained pipeline. The automated quantification showed an excellent agreement with manual scoring across the different behavioural responses, with minimal systematic bias. The pipeline reliably reproduced the expected increase in nocifensive behaviours following capsaicin administration, consistent with the results obtained from recordings generated at our institution. These results provide evidence that the pipeline maintains its performance when applied to data acquired independently and under different experimental conditions. Such inter‐laboratory reproducibility is particularly important for automated behavioural analysis, as differences in experimental settings, recording conditions and laboratory‐specific procedures can substantially affect the performance and transferability of computational tools.

The ability to quantify spontaneous behaviours with high temporal resolution provides new opportunities to evaluate analgesic efficacy beyond traditional withdrawal‐based paradigms, thereby enhancing the translational relevance of preclinical pain research. Moreover, recent advances in automated systems that standardize pain testing through computer‐controlled delivery of thermal, mechanical or optogenetic stimuli have further improved the precision and reproducibility of evoked pain assessments (Barkai et al. 2025; Black et al. 2020; Dedek et al. 2023; Zhang et al. 2022). Integrating such automation with behavioural machine learning approaches has the potential to accelerate the pace and reliability of analgesic drug discovery.

Despite these promising findings, several limitations should be acknowledged. First, the dataset was limited in size; although data augmentation and weighted sampling strategies partially mitigated this imbalance, future work should focus on expanding the training dataset to further improve model generalizability. Second, although the framework successfully generalized from the capsaicin training dataset to independent inflammatory (formalin) and cancer pain models and retained its ability to detect nocifensive responses in recordings independently generated at an external institution, the validation was limited to a single mouse strain. Future studies should therefore extend external validation across additional laboratories, recording configurations, mouse strains, sexes and chronic pain models, particularly neuropathic conditions in which spontaneous pain‐related behaviours may be subtler and more heterogeneous. Finally, while licking and flinching were selected as representative spontaneous nocifensive behaviours, the integration of additional behavioural measures (Bove et al. 2003), such as locomotor alterations, weight‐bearing asymmetry or affective behaviours, would provide a more comprehensive assessment of ongoing pain and further enhance the translational value of the platform.

In conclusion, this study provides a proof‐of‐concept for the use of deep learning in the automated quantification of spontaneous pain behaviours in mice. By reducing observer bias and enhancing throughput, our approach represents an important step towards standardized and clinically relevant assessment of nociception in preclinical research. Future refinements and the expansion of behavioural repertoires will further increase its value as a versatile platform for mechanistic studies and drug discovery in pain research.

## Author Contributions

This study was designed by M.Z., E.B., R.N., F.D.L., M.L., D.S.M.A. The experiments were performed by E.B. and G.D.S. D.S.M.A., L.P.G., M.L.Q.S. The data were analysed by M.Z., E.B., S.Q.K., D.S.M.A., and the results were critically examined by all authors. R.N., F.D.L., M.L. had a primary role in preparing the manuscript, which was edited by all authors. All authors have approved the final version of the manuscript and agree to be accountable for all aspects of the work.

## Conflicts of Interest

The authors declare no conflicts of interest.

## Supporting information


**Table S1:** Output labels (in seconds) of normal, licking and flinching behaviour.

**Video S1:** Labelled video reporting normal, licking and flinching behaviour.

## Data Availability

Data are available upon request from the corresponding author. The complete automated analysis pipeline (compiled as a standalone executable software with a GUI) along with the trained network weights and a detailed User Guide is publicly available on Zenodo at DOI: https://doi.org/10.5281/zenodo.21157073.
