# Supplementary material for: Deep Learning‐Based Quantification of Spontaneous Pain Behaviours in Mice
Source: Eur J Pain. 2026 Sep 23;30(9):e70391. doi: 10.1002/ejp.70391 (PMC13598732; doi:10.1002/ejp.70391)
Supplement: Supplementary file 1 [file EJP-30-0-s001.docx]

**Supplementary material**

**Supplementary video**. Labelled video reporting normal, licking, and flinching behavior.

**Supplementary** **table 1**. Output labels (in seconds) of normal, licking and flinching behavior.

| start time (seconds) | end time (seconds) | behaviour |
| --- | --- | --- |
| 0.00 | 1.16 | normal |
| 1.18 | 1.24 | flinching |
| 1.26 | 1.64 | normal |
| 1.66 | 1.82 | flinching |
| 1.84 | 2.34 | normal |
| 2.36 | 3.52 | licking |
| 3.54 | 3.70 | flinching |
| 3.72 | 4.44 | licking |
| 4.46 | 4.76 | flinching |
| 4.78 | 4.90 | licking |
| 4.92 | 4.94 | normal |
| 4.96 | 5.04 | licking |
| 5.06 | 6.04 | normal |
| 6.06 | 6.64 | licking |
| 6.66 | 6.80 | normal |
| 6.82 | 7.22 | flinching |
| 7.24 | 8.94 | licking |
| 8.96 | 9.50 | normal |
| 9.52 | 10.06 | flinching |
| 10.08 | 12.04 | licking |
| 12.06 | 12.32 | flinching |
| 12.34 | 13.48 | licking |
| 13.50 | 13.54 | normal |
| 13.56 | 14.94 | licking |
| 14.96 | 15.10 | flinching |
| 15.12 | 19.40 | licking |
| 19.42 | 20.40 | flinching |
| 20.42 | 20.42 | normal |
| 20.44 | 21.66 | licking |
| 21.68 | 22.50 | flinching |
| 22.52 | 22.56 | licking |
| 22.58 | 22.74 | normal |
| 22.76 | 23.58 | flinching |
| 23.60 | 29.42 | licking |
| 29.44 | 29.58 | flinching |
| 29.60 | 30.56 | licking |
| 30.58 | 30.64 | normal |
| 30.66 | 35.76 | licking |
| 35.78 | 36.10 | flinching |
| 36.12 | 36.32 | normal |
| 36.34 | 36.46 | flinching |
| 36.48 | 36.82 | normal |
| 36.84 | 36.96 | flinching |
| 36.98 | 37.24 | normal |
| 37.26 | 37.30 | flinching |
| 37.32 | 37.68 | normal |
| 37.70 | 38.20 | flinching |
| 38.22 | 38.64 | normal |
| 38.66 | 38.80 | flinching |
| 38.82 | 40.20 | normal |
| 40.22 | 40.48 | flinching |
| 40.50 | 40.62 | normal |
| 40.64 | 40.86 | flinching |
| 40.88 | 41.50 | normal |
| 41.52 | 41.58 | flinching |
| 41.60 | 41.92 | normal |
| 41.94 | 42.18 | flinching |
| 42.20 | 42.50 | normal |
| 42.52 | 42.54 | flinching |
| 42.56 | 42.86 | normal |
| 42.88 | 43.18 | flinching |
| 43.20 | 43.76 | normal |
| 43.78 | 43.82 | flinching |
| 43.84 | 44.22 | normal |
| 44.24 | 44.64 | flinching |
| 44.66 | 44.86 | normal |
| 44.88 | 45.10 | flinching |
| 45.12 | 45.14 | normal |
| 45.16 | 45.44 | flinching |
| 45.46 | 45.52 | licking |
| 45.54 | 45.58 | normal |
| 45.60 | 46.20 | flinching |
| 46.22 | 47.98 | licking |
| 48.00 | 49.20 | flinching |
| 49.22 | 49.40 | normal |
| 49.42 | 49.44 | flinching |
| 49.46 | 49.72 | normal |
| 49.74 | 49.82 | flinching |
| 49.84 | 49.92 | normal |
| 49.94 | 50.18 | licking |
| 50.20 | 50.40 | normal |
| 50.42 | 52.88 | licking |
| 52.90 | 53.18 | flinching |
| 53.20 | 57.16 | licking |
| 57.18 | 58.02 | normal |
| 58.04 | 58.22 | licking |
| 58.24 | 58.34 | normal |
| 58.36 | 58.54 | licking |
| 58.56 | 58.70 | normal |
| 58.72 | 59.14 | licking |
| 59.16 | 59.18 | normal |
| 59.20 | 60.00 | licking |
